# A window of opportunity: a pilot study exploring smoking cessation support during COPD hospitalisation

**DOI:** 10.3389/frhs.2025.1697187

**Published:** 2025-12-12

**Authors:** Ingeborg Farver-Vestergaard, Anders Løkke, Jannie Christina Frølund

**Affiliations:** 1 Department of Medicine, Lillebaelt Hospital, Vejle, Denmark; 2 Department of Regional Health Research, University of Southern Denmark, Odense, Denmark

**Keywords:** addiction, nicotine, implementation, respiratory disease, counseling

## Abstract

**Background:**

A significant proportion of patients with chronic obstructive pulmonary disease (COPD) continue smoking after diagnosis, contributing to increased symptom burden, more frequent exacerbations and poorer long-term outcomes. Hospitalisation due to COPD exacerbation may serve as a “window of opportunity” for delivering smoking cessation support.

**Aim:**

This pilot study evaluated the feasibility of integrating structured smoking cessation support into routine inpatient care for patients hospitalised with a COPD exacerbation.

**Methods:**

We followed 45 patients admitted for COPD exacerbation who reported active smoking at baseline. Smoking status and COPD symptoms (COPD Assessment Test, CAT) were evaluated at baseline, 1 month and 3 months after discharge. Comparisons were made between participants with smoking and non-smoking status at 1 month follow-up, and across three groups at 3 months: sustained non-smoking, sustained smoking and smoking relapse.

**Results:**

At 1 month, 30 patients (66.7%) reported abstinence, and 19 (42.2%) remained abstinent at 3 months. Improvements in mean CAT scores were observed over time, from 22.9 (95% CI = 20.0–25.7) at baseline to 13.9 (CI = 11.4–16.3) at 1 month and 12.9 (CI = 10.1–15.6) at 3 months. A trend towards lower CAT scores were observed for participants with non-smoking status at follow-up, compared with those who were smoking. We observed, that those who sustained non-smoking at follow-up were older, had higher baseline expectations of quitting and reported greater confidence in their ability to stop. However, those who relapsed at three months were the oldest. Being without a partner appeared more common among sustained smoking at follow-up.

**Conclusion:**

Smoking cessation support initiated during COPD hospitalisation was feasible and the majority of patients reported short-term abstinence and meaningful reductions in symptom burden. Age, expectations and confidence appeared to affect cessation trajectories, but should be explored further in larger, controlled trials and implementation setups.

## 1 Introduction

Smoking is the leading risk factor for the development and progression of chronic obstructive pulmonary disease (COPD) (1). Continued smoking after a COPD diagnosis contributes to increased symptom burden, accelerated disease progression, higher risk of exacerbation and elevated mortality risk (2). Despite these well-documented risks, approximately 35% of patients with COPD continue smoking after diagnosis (2).

Persistent smoking among patients with COPD is influenced by a range of complex factors. These include limited understanding of the disease and the ongoing harms of smoking post-diagnosis (3), feelings of stigma, guilt or shame that may lead patients to conceal their smoking or misreport their smoking status (4, 5), high levels of nicotine dependence due to long-term tobacco use (6) and psychosocial coping mechanisms centered around smoking (7). Although these barriers complicate cessation efforts, many patients with COPD express a desire to quit smoking but struggle to do so without support.

Hospitalisation, particularly during acute illness episodes such as COPD exacerbations, represents a “window of opportunity” to initiate health behaviour change (6, 8). This period often heightens patients' awareness of the consequences of smoking and stimulates their motivation to quit. Evidence from a recent Cochrane review shows that smoking cessation interventions initiated during hospitalisation and continued after discharge can significantly improve quit rates, with patients receiving such interventions being 36% more likely to stop smoking compared to those who received no inpatient support (8). However, despite the evidence supporting hospital-initiated cessation efforts, implementation remains inconsistent. Findings from our recent survey of hospital-based healthcare professionals suggest that smoking cessation support is often limited or absent at the hospital (9). Reported barriers include lack of time, inadequate training and uncertainty about how to counsel patients effectively (10).

On this background, the present pilot study aimed to assess the feasibility of integrating structured smoking cessation support into routine inpatient care for patients admitted with a COPD exacerbation. We evaluated smoking status and symptom levels over a three-month follow-up period. Moreover, we examined differences in patient characteristics and symptom levels based on smoking status at one and three months.

## 2 Methods

We conducted a prospective, single-arm pilot study of smoking cessation support initiated during COPD hospitalisation, using a descriptive, observational design with repeated measures over a 3-month follow-up period.

### 2.1 Setting and participants

The smoking cessation intervention was implemented at the Department of Medicine, Vejle Hospital, Denmark, beginning in January 2024. All patients admitted with an acute exacerbation of COPD during the evaluation period January to December 2024 were considered for inclusion in the present evaluation study.

Patients were eligible for inclusion if they (1) had a confirmed diagnosis of COPD, (2) were hospitalised due to a COPD exacerbation, (3) reported current smoking at the time of admission, and (4) were able to understand and complete written questionnaires in Danish. Patients with cognitive or language barriers preventing questionnaire completion were excluded. Eligible patients were invited to participate by the designated project nurse and asked to complete questionnaires at baseline (during hospitalisation), and again at one and three months after discharge.

All participants provided written informed consent. The Regional Committees on Health Research Ethics for Southern Denmark waived the need for formal ethical approval for this project (S-20242000-127).

### 2.2 Intervention

The smoking cessation intervention was integrated in routine inpatient care and delivered by a nurse as part of her usual work in the clinic. The intervention consisted of the following key components:

- Counseling: The nurse delivering the intervention received two hours of structured training in evidence-based pharmacological, motivational and behavioural smoking cessation counseling. This was based on principles from Motivational Interviewing (11) and the Stages of Change framework (12)
- Pharmacotherapy: Patients were offered either nicotine replacement therapy (NRT) or cytisine, based on their preference and the clinical appropriateness. Treatment decisions were made collaboratively, with input from a respiratory physician when needed.
- Post-discharge referral and follow-up: The nurse followed up on the patients' smoking status and needs at one and three months after discharge. Patients in need of further support were referred to community-based smoking cessation programmes.

To support intervention delivery, the team developed written materials and podcasts, including:

- A patient leaflet outlining the health risks of continued smoking and the benefits of cessation in COPD.
- A patient-centred podcast designed to provide accessible information and motivation for smoking cessation (13, 14).
- A shared decision-making tool to facilitate personalised discussions about advantages and disadvantages of smoking cessation and continued smoking.

The intervention was delivered flexibly during the hospitalisation and was tailored to individual patient needs. Sessions were brief and informal, with repeated contact depending on the length of stay.

### 2.3 Data collection

Patients completed self-administered questionnaire at three time points: during hospitalisation (baseline), and at one and three months post-discharge. If patients required assistance due to health or literacy challenges, the project nurse provided support to facilitate accurate questionnaire completion without influencing responses. Data collection included (a) sociodemographic and clinical information, (b) smoking history and smoking status, (c) motivation and perceived importance and competencies for cessation and (d) COPD symptoms and impact on everyday life, assessed using the COPD Assessment Test (CAT) (15). The CAT consists of eight items assessing symptoms and functional impact: cough, sputum production, chest tightness, breathlessness during physical activity, functional limitations at home, functional limitation outside the home, sleep, and energy level. Each item is rated on a 0–5 scale, yielding a total score from 0 to 40, with higher scores reflecting more severe COPD impact.

### 2.4 Statistical analysis

Descriptive statistics were used to summarise baseline characteristics, smoking history, cessation motivation and CAT scores. The normality of continuous variables was evaluated using graphical methods (histograms and Q–Q plots) and the Shapiro–Wilk test. Smoking status was assessed at baseline, as well as one and three months after discharge, and point-prevalence abstinence rates were calculated at each follow-up point.

To examine associations between smoking status and patient characteristics, appropriate statistical tests were applied based on data distribution. Normally distributed continuous variables were compared using *t*-tests or one-way ANOVA, whereas non-normally distributed variables were analysed using Mann–Whitney *U* or Kruskal–Wallis tests. We used parametric or non-parametric tests as appropriate. Categorical variables were compared using chi-squared or Fisher's exact tests as appropriate.

At one month, participants were classified as either non-smoking vs. smoking. At three months, participants were categorised as:

- Sustained non-smoking: reporting non-smoking at both follow-ups
- Sustained smoking: reporting smoking at both follow-ups
- Smoking relapse: reporting non-smoking at one month but smoking at three months

These groups were compared in terms of sociodemographic, clinical, smoking history, cessation motivation and CAT score data at baseline and follow-up.

Due to the exploratory nature of the study and limited sample size, no formal hypothesis testing was performed. All analyses were conducted using Stata version 18 (StataCorp LLC, College Station, TX, USA).

## 3 Results

A total of 45 patients hospitalised for a COPD exacerbation and reporting active smoking at admission were included in the study. All but two patients (95.6%) started pharmacotherapeutic cessation treatment during hospitalisation. All patients, except five (88.9%), were referred to a community-based smoking cessation programme upon discharge. Demographic and clinical characteristics of participants at baseline are presented in Table 1.

**Table 1:** Patient characteristics at baseline (*N* = 45).

Patient characteristics	Mean/Median/*n*	SD/IQR/%	Range
Age (median, IQR)	69	60–76	31–87
Female (*n*, %)	27	60.0%	–
Relationship status (*n*, %) (missing = 1)			
Married/relationship	21	47.7%	–
Alone/no steady relationship	23	52.3%	–
Co-habitation status (*n*, %) (missing = 1)			
Living alone	24	54.6%	–
Living with one or more others	20	45.4%	–
Lung function, FEV1% pred. (*n*, %) (missing = 11)			
<30%	7	20.6%	–
30%–50%	11	32.4%	–
50%–80%	8	23.5%	–
>80%	8	23.5%	–
CAT total score (mean, SD) (missing = 1)	22.9	9.4	2–39
How important is smoking cessation for you? NRS 0–10 (median, IQR) (missing = 16)	10	7–10	0–10
How confident are you that you can quit smoking? NRS 0–10 (median, IQR) (missing = 16)	8	5–10	0–10
Expect to be smoke-free in one month (*n*, %) (missing = 0)	33	73.3%	–
Expect to be smoke-free in 6 months (*n*, %) (missing = 1)	43	97.7%	–
Expect to be smoke-free in 12 months (*n*, %) (missing = 1)	43	97.7%	–
Age at smoking debut (median, IQR) (missing = 0)	14	13–16	7–40
Partner smoking (*n*, %) (missing = 0)	14	31.1%	–

CAT, COPD Assessment Test, higher scores indicating more severe symptpoms; FEV1% pred., Forced Expiratory Volume in 1 s, percentage of predicted, higher scores indicating better lung function; IQR, inter quartile range; SD, Standard deviation.

### 3.1 Smoking status and COPD symptoms over time

**Figure 1:**
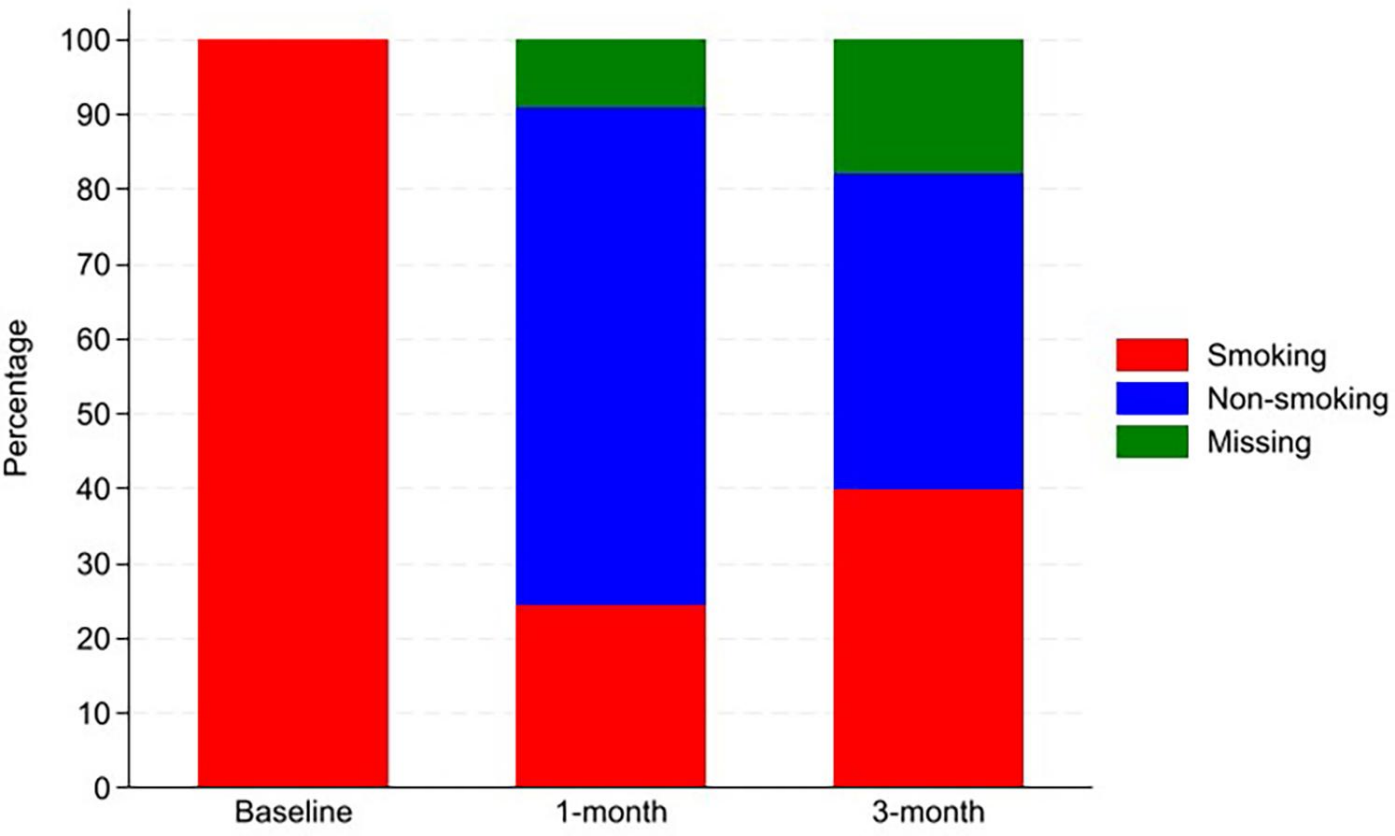
Smoking status at baseline as well as at one and during COPD hospitalisation (*N* = 45).

At one month follow-up, 30 patients (66.7%) reported non-smoking while 19 patients (42.2%) sustained non-smoking at three months (Figure 1).

Reductions in CAT scores were observed over time in the overall sample with confidence intervals that did not overlap across time points (Table 2).

**Table 2:** COPD symptoms [measured with the COPD assessment test (15)] at baseline as well as at one and three months follow-up after smoking cessation support during COPD hospitalisation (*N* = 45).

COPD assessment test- score	Missing	Mean	SE	95% CI
Baseline	1	22.9	1.42	20.0–25.7
1-month follow-up	5	13.9	1.22	11.4–16.3
3-month follow-up	8	12.9	1.36	10.1–15.6

COPD, Chronic obstructive pulmonary disease; SE, Standard error; CI, Confidence interval.

### 3.2 Descriptive comparison of baseline characteristics by smoking status

At one-month follow-up (Table 3), participants with non-smoking status were significantly older than those with smoking status and more commonly expected to be smoke-free when they were asked at baseline. There was a trend toward females being more likely to report smoking at the one month follow-up. Differences in remaining variables did not reach statistical significance.

**Table 3:** Characteristics of participants who reported smoking vs. non-smoking at 1-month follow-up after smoking cessation support during COPD hospitalisation (*n* = 45).

Patient characteristics	Smoking (*n* = 11)		Non-smoking (*n* = 30)		*p*
	Mean/Median/*n*	SD/IQR/%	Mean/Median/*n*	SD/IQR/%	
Age (median, IQR) (missing = 4)	60	44–71	71	67–76	*0*.*04*
Sex (*n*, %)					0.08
Male	2	18.2%	15	50.0%	
Female	9	81.8%	15	50.0%	
Lung function, FEV1% pred. (*n*, %)					0.50
<30%	3	27.3%	3	10.0%	
30%–50%	2	18.2%	7	23.3%	
50%–80%	1	9.1%	7	23.3%	
>80%	2	18.2%	5	16.7%	
CAT total score at baseline (mean, SD)	24.1	3.5	22.9	1.6	0.73
CAT total score at one month follow-up (mean, SD)	14.6	9.9	13.6	6.9	0.70
Age at smoking debut (median, IQR)	14.5	2.8	15.7	5.7	0.70
Partner smoking (n, %)					0.16
Yes	2	18.1%	11	36.7%	
No	1	9.1%	8	26.7%	
Do not have a partner	8	72.8%	11	36.7%	
Expected at baseline to be smoke-free in one month (n, %)					*0*.*01*
Yes	5	45.5%	26	86.7%	
No	6	54.5%	4	13.3%	
Perceived importance of smoking cessation at baseline, NRS 0–10 (median, IQR)	8	6–10	10	8.5–10	0.11
Perceived confidence in ability to stop at baseline, NRS 0–10 (median, IQR)	8	5–10	8	6–10	0.65

CAT, COPD Assessment Test, higher scores indicating more severe symptoms; COPD, Chronic obstructive pulmonary disease; FEV1% pred., Forced Expiratory Volume in 1 s, percentage of predicted, higher scores indicating better lung function;, IQR, Inter quartile range;, NRS, Numeric rating scale; SD, Standard deviation.

Italics indicate statistically significant *p*-values of <0.05.

At three months (Table 4), statistically significant differences in age were observed among the groups with sustained non-smoking, sustained smoking and smoking relapse. Participants who had maintained abstinence (sustained non-smoking) were older than those who continued smoking across both follow-up points (sustained smoking), but younger than those who relapsed to smoking between one and three months (smoking relapse). A near-significant trend was observed for CAT scores at three months, with the sustained non-smoking group reporting markedly lower symptom levels compared with the sustained smoking and smoking relapse groups. Differences in remaining variables were not statistically significant.

**Table 4:** Characteristics of participants who sustained non-smoking, sustained smoking and relapsed to smoking at three months follow-up after smoking cessation support during COPD hospitalisation (*n* = 45).

Patient characteristics	Sustained non-smoking		Sustained smoking		Smoking relapse		*p*
	Mean/Median/*n*	SD/IQR/%	Mean/Median/n	SD/IQR/%	Mean/Median/*n*	SD/IQR/%	
*N*, % (missing = 11)	18	40%	8	11%	8	11%	–
Age (median, IQR)	71	67–75	51.5	43–64.5	76	70.5–80	*0*.*002*
Sex (*n*, %)							0.13
Male	8	44.4%	2	25.0%	6	75.0%	
Female	10	55.6%	6	75.0%	2	25.0%	
Lung function, FEV1% pred. (n, %)							0.58
<30%	1	5.6%	3	37.5%	1	12.5%	
30%–50%	5	27.8%	1	12.5%	1	12.5%	
50–80%	5	27.8%	1	12.5%	1	12.5%	
>80%	3	16.7%	2	25.0%	1	12.5%	
CAT total score at baseline (mean, SD)	22.6	8.6	22.7	11.5	21.8	7.1	0.97
CAT total score at three months follow-up (mean, SD)	17.3	6.7	25.0	11.5	21.9	4.6	0.06
Age at smoking debut (median, IQR)	15	14–16	14	13–16.5	14	12–15	0.13
Partner smoking (n, %)							0.62
Yes	6	33.3%	1	12.5%	2	25.0%	
No	5	27.8%	1	12.5%	2	25.0%	
Do not have a partner	7	38.9%	6	75.0%	4	50.0%	
Expected at baseline to be smoke-free in one month (n, %)							0.11
Yes	16	88.9%	4	50.0%	6	75.0%	
No	2	11.1%	4	50.0%	2	25.0%	
Expected at baseline to be smoke-free in six months (*n*, %) (missing = 1)							-
Yes	18	100.0%	8	100%	7	87.5	
No	0	0.0%	0	0.0%	0	0.0%	
Perceived importance of smoking cessation at baseline, NRS 0–10 (median, IQR)	10	10–10	8	7–10	10	8–10	0.50
Perceived confidence in ability to stop at baseline, NRS 0–10 (mean, SD)	10	8–10	8	5–10	7	5–8	0.19

CAT, COPD Assessment Test, higher scores indicating more severe symptoms; COPD, Chronic obstructive pulmonary disease; FEV1% pred., Forced Expiratory Volume in 1 s, percentage of predicted, higher scores indicating better lung function; IQR, Inter quartile range; NRS, Numeric rating scale; SD, Standard deviation.

Italics indicate statistically significant *p*-values of <0.05.

## 4 Discussion

We found that patients who received smoking cessation support during hospitalisation for COPD achieved abstinence rates of 66.7% at one month and 42.2% at three months after discharge. These rates are slightly higher than the abstinence rates generally reported in smoking cessation trials [around 50%–60%, but decreasing within the first three months (16)]. Notably, the abstinence rates observed in our study exceed those reported in a recent study where smoking cessation support was provided for patients with COPD immediately in conjunction with an outpatient hospital consultation (17). In that outpatient study, access to smoking cessation medication was the only significant predictor of cessation success, yet only 69.3% of patients in the intervention group had access to such medication. In contrast, 95.6% of patients in our study received medication support, which may partly explain the higher cessation rates observed. These findings support our initial assumption that hospitalisation represents a window of opportunity for smoking cessation interventions, which could be complemented by ongoing support at other points along the patient care trajectory.

Beyond abstinence rates, we observed improvements in COPD-related health status. COPD symptoms, measured by the CAT, tended to decrease over time. On average, patients improved by 9–10 points, corresponding to a shift from high to medium disease impact (18). While some of this improvement may reflect recovery from exacerbation, we observed a near-significant trend towards markedly lower symptom levels in the group of sustained non-smoking at three months follow-up, compared with those reporting smoking relapse. Hence, smoking cessation appeared to contribute to better symptom control, but these findings should be explored further in larger samples.

Subgroup analyses revealed additional insights into potential confounders that should be explored further in future large-scale, controlled and/or implementation studies. At one month, participants who reported non-smoking tended to be older than those who reported smoking, but at three months, those who relapsed to smoking were the oldest group. This suggests that while older patients may initially be motivated—possibly due to longer illness histories or greater symptom burden—they may still be at risk of relapse. Existing studies, as well as theories of behaviour change (19, 20), also emphasise that repeated attempts and accumulated experience are part of the cessation process, which could help explain these patterns.

Trends in sex differences were less consistent. A larger proportion of males reported non-smoking at one month, but males were also more likely to report smoking relapse by three months. However, these findings did not reach statistical significane in the present pilot study. Expectations of quitting could potentially be relevant, as most of the participants who sustained non-smoking at follow-up had expected to be smoke-free during hospitalisation. Interestingly, a majority of those who relapsed to smoking had also anticipated success, which could indicate that motivation alone is insufficient. All patients had expected to be smoke-free within six months, which—again—shows that even optimistic cases can find it hard to maintain abstinence. This aligns with evidence that sustained abstinence typically requires multiple attempts (19). Relapse at three months should therefore not be regarded as failure, but as part of a longer-term process that benefits from continued support.

We observed that those who sustained non-smoking reported higher baseline confidence in their ability to quit, albeit non statistically significant. If this finding is confirmed in larger studies it could suggest that early assessment of confidence may help identify those in need of additional encouragement and tailored support. Social context could play a role as well: participants who sustained smoking across follow-up points were observed to more often lack a partner, underscoring the importance of social support and structured follow-up when patients transition from hospital to home.

Taken together, these findings highlight that smoking cessation after COPD hospitalisation is not a binary outcome but a dynamic process with multiple trajectories. Some patients maintain abstinence, some relapse, and others continue smoking, each group appearing to be characterised by different baseline profiles. These trends can be used to generating hypotheses for testing in future, larger scale studies. Recognising and exploring these patterns further can potentially help to inform the design of more personalised and effective interventions.

The intervention in this study was based on a combination of components that have been shown to lead to increased likelihood of cessation among patients with COPD (21): pharmacotherapy, motivational counseling and behavioural support. It was delivered in a flexible manner by a nurse as part of her usual tasks in the clinic. The hospital setting, which often involves several days of admission, provided time to build trust, tailor conversations and gradually reinforce motivation. This context may explain the relatively high cessation rates. Nonetheless, cessation is rarely achieved in a single attempt. Each quit effort should be viewed as a step forward, and healthcare systems should provide ongoing, cross-sectoral support to sustain progress. Importantly, smoking cessation should be recognised not only as prevention but also as an important treatment strategy for COPD, with the potential to improve symptoms, prognosis and quality of life (16).

### 4.1 Limitations

This was a non-controlled pilot study, so beneficial effects may partly reflect confounding factors, which should be explored further in large-scale studies. The pilot design and the fact that the intervention was delivered by a single nurse limit generalisability of the findings, and also means that we cannot determine which specific components were most effective. Abstinence outcomes were based on self-report without biochemical validation, raising potential reporting bias, although prior evidence suggests self-reports are generally reliable in older populations (22).

## 5 Conclusions

Smoking cessation support initiated during hospitalisation for COPD was feasible and we observed a relatively high short-term abstinence rates after the intervention along with meaningful, near-significant improvements in symptom burden. Patients' age, self-efficacy and expectations appeared to influence cessation trajectories. Hence, these effects, potential confounders along with mediating factors should be tested in large-scale, controlled trials.

## Data Availability

The raw data supporting the conclusions of this article will be made available by the authors, without undue reservation.
